# Somatic embryogenesis and enhanced shoot organogenesis in *Metabriggsia ovalifolia* W. T. Wang

**DOI:** 10.1038/srep24662

**Published:** 2016-04-19

**Authors:** Yao Ouyang, Yulu Chen, Jinfeng Lü, Jaime A. Teixeira da Silva, Xinhua Zhang, Guohua Ma

**Affiliations:** 1 Key Laboratory of Plant Resources Conservation and Sustainable Utilization, South China Botanical Garden, the Chinese Academy of Sciences, Guangzhou, 510650, China; 2 University of Chinese Academy of Sciences, Beijing 100039, China; 3 P.O. Box 7, Miki-cho Post Office, Miki-cho, Ikenobe 3011-2, Kagawa-ken, 761-0799, Japan

## Abstract

An efficient protocol providing a dual regeneration pathway via direct shoot organogenesis and somatic embryogenesis for an endangered species, *Metabriggsia ovalifolia* W. T. Wang, was established from leaf explants. When applied at 2.5 μM, the cytokinins 6-benzyladenine (BA) or thidiazuron (TDZ) and the auxins indole-3-butyric acid (IBA), α-naphthaleneacetic acid (NAA) and indole-3-acetic acid (IAA) could induce shoots when on basal Murashige and Skoog (MS) medium. BA and TDZ could induce more adventitious shoots (19.1 and 31.2/explant, respectively) than NAA (4.6/explant), IBA (5.7/explant) or IAA (6.4/explant). BA and TDZ at 5–10 μM could induce both shoots and somatic embryos. A higher concentration of TDZ (25 μM) induced only somatic embryos (39.8/explant). The same concentration of BA induced both adventitious shoots (23.6/explant) and somatic embryos (9.7/explant). Thus, somatic embryogenesis in this plant needs a high cytokinin concentration (BA; TDZ), as evidenced by histology. Somatic embryos germinated easily when left on the same media, but formed adventitious roots in two weeks on MS supplemented with 0.5 μM NAA, 0.5 μM IBA and 0.1% activated charcoal. Over 93% of plantlets survived following acclimatization and transfer to a mixture of sand and vermiculite (1:1, v/v) in trays.

*Metabriggsia ovalifolia* W. T. Wang (Gesneriaceae) is a perennial herb that is exclusively endemic to Guangxi and Yunnan provinces in China^1^,^2^ and is one of four species in the Gesneriaceae that has been listed as “first grade rare and endangered species in China”^3^,^4^. The establishment of an efficient propagation and plant regeneration system is one effective way of circumventing sudden deterioration or loss of a plant’s natural environment, and thus serves as an ideal tool to preserve and utilize this rare and endangered plant species. Previously, a protocol for shoot organogenesis from leaf explants was reported for *M. ovalifolia*^5^. However, to date, no report exists on somatic embryogenesis for this plant. The main focus of this study was to assess the ability of different plant growth regulators (PGRs) to control somatic embryogenesis and shoot organogenesis in *M. ovalifolia* in a bid to expand the choices for viable *in vitro* regeneration pathways which would make applied biotechnological applications, such as genetic transformation, feasible.

## Materials and Methods

As described in Ma *et al*.^5^, *M. ovalifolia* plants growing in the high mountain areas of Hechi, Guangxi Province, at 600–1200 masl, were brought back alive and intact to the alpine and polar conservator of South China Botanical Garden (SCBG), Guangzhou. Plants were transplanted into several basins (20 cm in height; 30 cm in diameter) containing sand, ocher and humus soil (1:1:1, w/w) in March, 2009. Using the protocol of Yang *et al*.^6^, “healthy leaves were surface sterilized in 70% (v/v) alcohol for 10 s and in 0.1% (w/v) mercuric chloride for 8 min, rinsed with sterile distilled water three times, then cut into 1.0 cm^2^ explants”, which were inoculated on MS basal medium^7^ supplemented with 5.0 μM 6-benzyladenine (BA) and 0.5 μM α-naphthaleneacetic acid (NAA). After culturing for 2 weeks in the dark, the cultures were transferred to light culture, and callus (with adventitious shoots) clumps were transferred to the same medium for subculture once every two months. All the culture jars (200 ml) were 10 cm high and 6 cm in diameter. All media contained 30 g l^−1^ sucrose and were adjusted to pH 5.8 and solidified with 0.6% agar (Sigma-Aldrich, St. Louis, USA) prior to autoclaving. All cultures were placed under light conditions with a 10-h photoperiod at a photosynthetic photon flux density of 80 μmol m^−2^ s^−1^ (with two cool white fluorescent lamps, 40 Watts each) and cultured at 25 ± 2 °C 5. Immature leaves (about 0.4 cm^2^ in size) were used as explants for ensuing experiments.

### Effect of plant growth regulators on morphogenesis

Immature *in vitro* leaves (about 0.4 cm^2^ in size) were used as initial explants. The leaves were inoculated abaxial side down onto MS medium supplemented with the same concentration (2.5 μM) of PGRs for morphogenic induction (Table 1). Among the PGRs tested, thidiazuron (TDZ), indole-3-acetic acid (IAA) and zeatin (ZEA) were filter (0.24 μm) sterilized and added after autoclaved medium had cooled while 2,4-dichlorophenoxyacetic acid (2,4-D), BA, indole-3-butyric acid (IBA), kinetin (KIN), and NAA (Sigma-Aldrich, St. Louis, USA) were added prior to autoclaving at 121 °C for 15 min. Separately, the same leaf explants were inoculated onto MS medium supplemented with different concentrations (2.5–25 μM) of BA and TDZ, respectively (Table 2). All treatments consisted of 30 explants equally divided between five jars that were placed for 2 weeks in the dark then transferred to light culture under conditions described above. After a total of 5 weeks’ culture, morphogenesis (shoot organogenesis or somatic embryogenesis) was investigated. Experiments were repeated three times.

### Impact of a high concentration of TDZ on somatic embryogenesis and plant recovery

Immature leaves, which were used as explants, were plated abaxial surface down onto MS medium supplemented with a high concentration (25 μM) of TDZ. Every jar contained only one leaf explant. Culture jars were placed in the dark for 2 weeks and then transferred to light culture under the same conditions indicated above. After a total of 5 weeks of culture, somatic embryogenesis was investigated. After another 3 weeks of light culture, plant recovery from somatic embryos was investigated.

### Histological investigation of somatic embryogenesis of *in vitro*-grown leaf explants

This protocol follows Yang *et al*. (2013) precisely. “To study the ontogeny and development of embryogenic cell masses and somatic embryos, leaf explants incubated on induction medium containing 5.0 μM and 25.0 μM TDZ were subjected to histological analysis. The culture jars were cultured in the dark for 20 days then transferred to low-light intensity (<10 μmol m^−2^ s^−1^). After culturing for 0, 3, 4, 5, 6 and 7 weeks, the leaves (or callus clumps) were fixed in FAA (1:1:18, formaldehyde: glacial acetic acid: 70% alcohol) solution and maintained at room temperature for one week, then transferred to 70% alcohol for storage until required for analysis. The samples were stained with hematoxylin at first, then dehydrated in an increasing alcohol series (35%, 50%, 75%, 85%, 95% and pure alcohol), and embedded in paraffin. Transverse sections 8 μm thick were made with a paraffin-compatible microtome (Fourth Shanghai Medicine Manufacturing Co., Shanghai, China) and then laid on filter paper with a brush. Sequential sections were placed in parallel on a slide with 0.1% formalin at 40–45 °C to allow the sections to stretch. Slides were then placed in a warm box at 60 °C. The sections were dewaxed with xylene three times and finally covered with neutral balsam. Sections were observed under a microscope (Olympus SZX12)” (Yang *et al*. 2013).

### Shoot proliferation

Adventitious shoot clusters, each holding 8–10 shoots, were transferred to MS medium with 5.0 μM BA and 0.5 μM NAA, cultured in the light and subcultured on the same medium every 40 days.

### Formation of roots and acclimatization of recovered somatic embryos and adventitious shoots

After leaf explants were cultured on MS medium supplemented with a high concentration of TDZ (25 μM) for a total of 8 weeks, the germinated somatic embryos were isolated and transferred to half-strength MS medium containing 0.5 μM NAA, 0.5 μM IBA and 0.1% activated charcoal to induce roots^5^. When plantlets were 45 days old, and had 4–5 leaves and a height of 4 cm, they were removed from jars and agar was gently rinsed off in water. To induce roots, single shoots were excised from multiple shoot clusters and inserted into rooting medium containing 0.5 μM IBA and 0.2% activated carbon and incubated in the light. After 50 days, plantlets’ roots were washed free of agar. In total, 400 plantlets were transplanted to three trays containing potting mixture (sand: vermiculite; 1:1, w/w) under 50% shading in an open-air shed where they were watered once a day. Each plantlet was provided with 2 ml of half-strength MS (MS micronutrients only) once a week. Plant survival was quantified after 4 weeks. Three months later, plantlets were transferred to a substrate of sand, ocher and humus soil (1:1:1, w/w) in clay basins (20 cm in height; 30 cm in diameter) and maintained under shade under natural conditions.

### Statistical analyses

Statistical analyses followed Lü *et al*.^8^. “Experiments were repeated three times within a two-week interval. Each experimental treatment contained six explants per culture jar (separate jars for different explants) and five jars per treatment. Data was separated by one-way analysis of variance (ANOVA) and treatment means were considered to be significantly different from controls by the Least Significant Difference (LSD) test at *P* ≤ 0.05 using SPSS v. 18.0” (IBM, USA).

## Results

### Effect of plant growth regulators on morphogenesis

**Figure 1:**
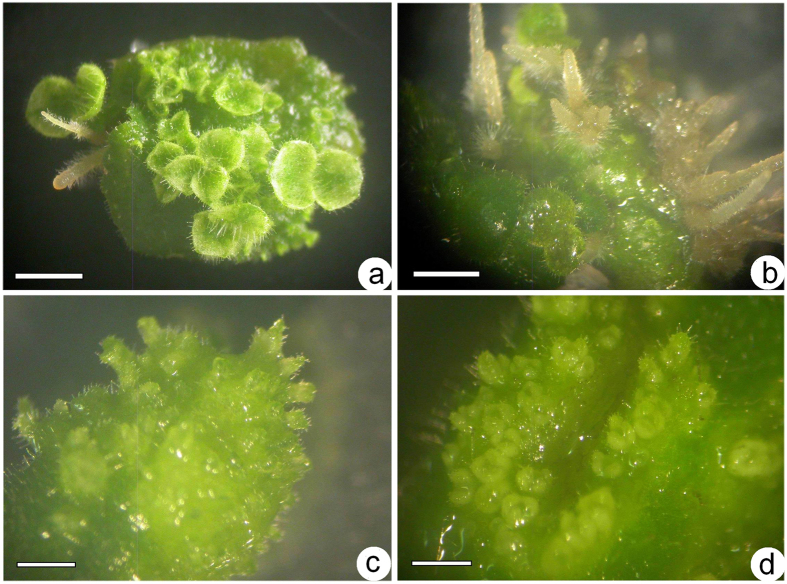
Shoot organogenesis on induction media supplemented with the same concentration (2.5 μM) of a single PGR from leaf explants of *Metabriggsia ovalifolia* after culture for 5 weeks (bars = 2 mm). Adventitious shoots and roots developed from an immature leaf explant on induction medium containing 2.5 μM IAA (**a**) or 2.5 μM NAA (**b**). Adventitious shoots developed from an immature leaf explant on induction medium containing 2.5 μM BA (**c**) or 2.5 μM TDZ (**d**).

Different PGRs, including auxins (2,4-D, IBA, IAA and NAA) and cytokinins (BA, KT, TDZ and ZEA), used at the same concentration (2.5 μM), were used to induce shoot organogenesis from leaf explants. The control consisted of no PGRs. This protocol, which was similar to the report by Ma *et al*.^5^, allowed for reproducibility to be tested, and also included ZEA as a novel factor. No growth was observed within the first 4 weeks from leaf explants on PGR-free medium. By the seventh week, a few small protuberances with limited callus formed on the leaf surface or on cut surfaces, some of these developing into adventitious shoots (Table 1). Callus, which was induced on the cut surface of leaf explants within 2–3 weeks on MS medium with 2.5 μM 2,4-D, could not develop shoots and became necrotic after browning. Leaf explants remained green for 2–3 weeks in the presence of 2.5 μM IAA, forming some protuberances and adventitious shoots directly on the leaf surface or on cut surfaces and adventitious roots 2 weeks later (Fig. 1a). Callus clusters and some adventitious roots could be induced in the presence of 2.5 μM NAA or 2.5 μM IBA within 2 weeks and adventitious shoots within 5 weeks (Fig. 1b). The use of 2.5 μM of KIN or ZEA caused leaf explants to turn yellow and necrotic, and no adventitious shoots formed. Initially (first 2 weeks), leaf explants were unresponsive to 2.5 μM BA, but by the fourth week, swollen explants formed protuberances on the leaf lamina with clearly formed adventitious shoots by the fifth week (Fig. 1c). Leaf explants were initially unresponsive to 2.5 μM TDZ, swelled within 2 weeks, and formed callus on cut surfaces and on the lamina, and by the fifth week, adventitious shoots were clearly visible (Fig. 1d). Among all PGRs supplied at the same concentration (2.5 μM), TDZ induced the most adventitious shoots, followed by BA (Table 1). Somatic embryogenesis was never observed in any of these media.

### Effect of different concentrations of BA and TDZ on shoot organogenesis and somatic embryogenesis

**Figure 2:**
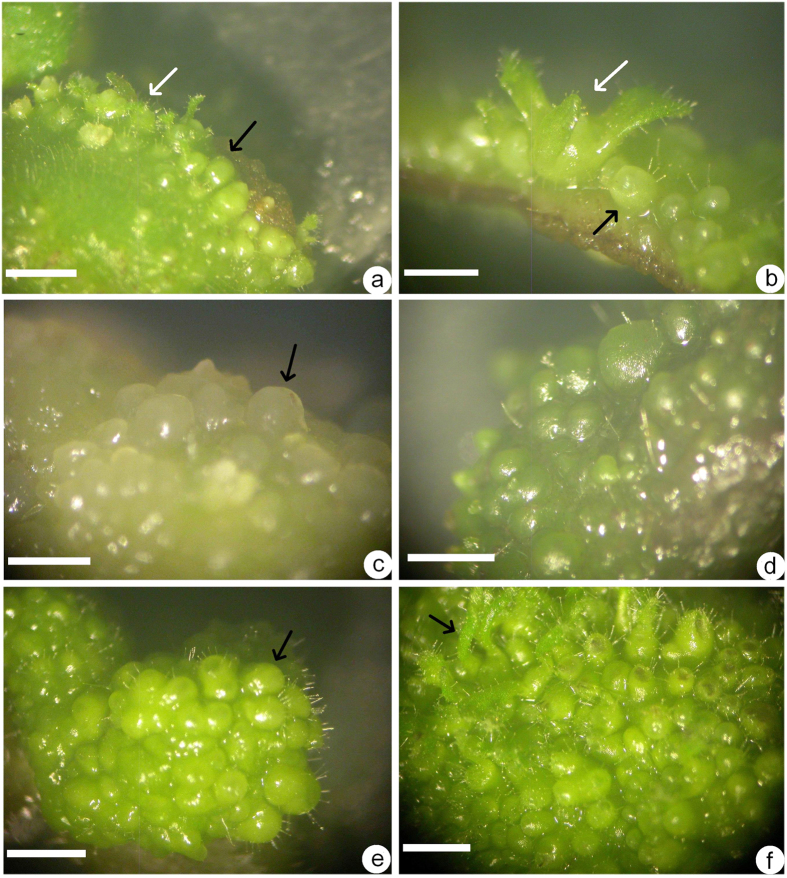
Shoot organogenesis and somatic embryogenesis in *Metabriggsia ovalifolia*. (**a**) Adventitious shoots (white arrow) and somatic embryos (black arrow) induced from an immature leaf explant on induction medium containing 10 μM BA after culture for 5 weeks. (**b**) Adventitious shoot and some globular- and torpedo-shaped somatic embryos visible on the surface of a leaf explant on induction medium containing 10 μM BA after culture for 6 weeks. (**c**) Some yellow protuberances (black arrow) occurred on the leaf surface on induction medium containing 10 μM TDZ after culture for 4 weeks. (**d**) Some globular somatic embryos were visible on the surface of leaf explants on induction medium containing 10 μM TDZ after culture for 5 weeks. (**e**) Somatic embryos induced on a leaf explant on induction medium containing 25 μM TDZ after culture for 6 weeks. (**f**) Some somatic embryos germinated and developed epicotyl directly on induction medium containing higher concentration of TDZ (25 μM) after culture for 7 weeks. Bars = 2 mm.

**Figure 3:**
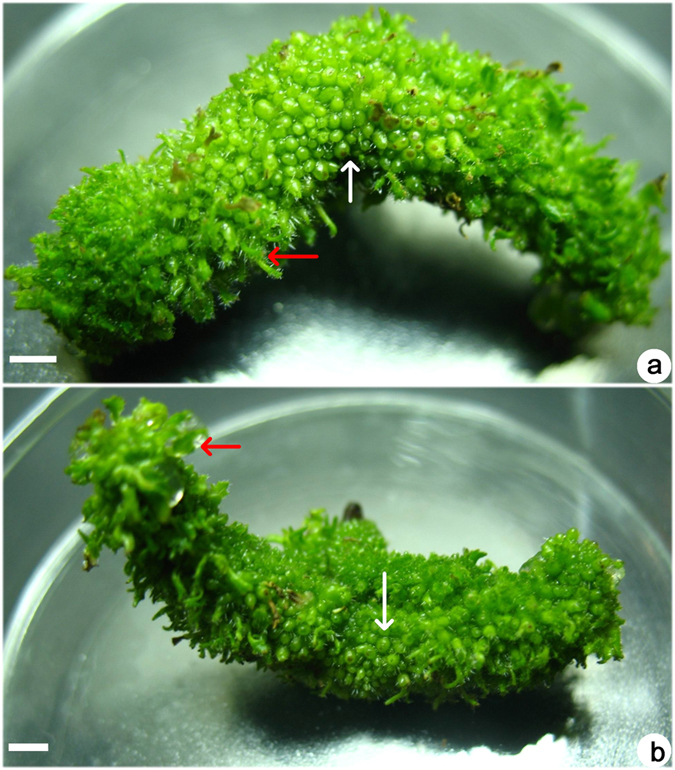
A high concentration (25 μM) of TDZ induced somatic embryogenesis and recovery of somatic embryos on the same leaf explant (one side (**a**) and the opposite side (**b**)) of *Metabriggsia ovalifolia*. Somatic embryos germinated first from both extremities of the leaf explant or from tissue that was in contact with the medium (red arrows) while the somatic embryos in the center of the explant remained in a non-germinated state at the globular stage (white arrows). Bars = 0.5 cm.

When different concentrations of BA or TDZ were added to MS medium, some trends were observed. Firstly, a low concentration (1.0–2.5 μM) of BA or TDZ showed the results described in the above section, i.e., exclusive shoot organogenesis with five weeks of culture. Secondly, as BA concentration increased to 5–25 μM, some adventitious shoots and somatic embryos formed on the surface of the leaf explants, i.e., mixed organogenesis. Globular and torpedo-shaped somatic embryos were observed on the surface of leaf explants (Fig. 2a,b). As BA concentration increased, the frequency of somatic embryogenesis also increased (Table 2). Thirdly, as the TDZ concentration was increased to 5–25 μM, somatic embryos formed easily on leaf explants, and the frequency of somatic embryogenesis increased (Table 2). At first, some yellow protuberances formed within 2–3 weeks (Fig. 2c), and when cultures were transferred to light, these protuberances became green and globular (Fig. 2d). Prolonging the culture period resulted in an increasing number of globular and torpedo stage somatic embryos (Fig. 2e). After culture for seven weeks, some somatic embryos germinated directly and developed a shoot tip at the top of the somatic embryo (Fig. 2f). As the TDZ concentration increased to 25 μM, only somatic embryogenesis was induced and adventitious shoots were not visible (Fig. 2e,f) with a single leaf explant inducing as many as hundreds of somatic embryos (Fig. 3a,b). However, the total number of somatic embryos decreased (Table 2). Somatic embryos located on both sides of the leaf explants or in contact with the medium developed earlier than the middle parts. The former germinated early, forming a well-developed shoot tip while the somatic embryos in the middle part remained at the globular or torpedo-shaped stage (Fig. 3a,b).

How then to distinguish adventitious shoots from somatic embryos? At an earlier stage, adventitious shoots and somatic embryos all developed from yellow protuberances. The adventitious shoots usually developed leaves at first and then shoots developed sequentially after. In the case of somatic embryos, there were distinctly visible stages, with globular and torpedo stages being most discernable. As the somatic embryo germinated, epicotyl (like an apical shoot) developed at first, and then leaves developed sequentially after.

### Histology of somatic embryogenesis from *in vitro* leaves

**Figure 4:**
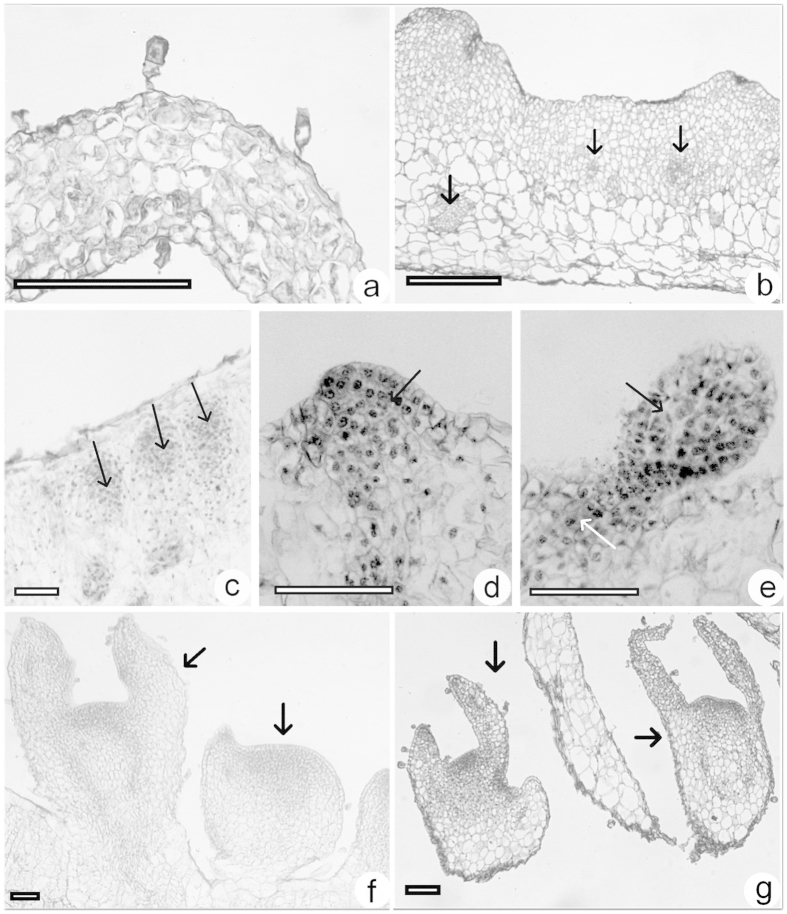
Light microscopic sections of somatic embryogenesis on induction medium supplemented with 5 μM and 25.0 μM TDZ from *in vitro* leaf explants of *Metabriggsia ovalifolia*. (**a**) Fresh leaf section. (**b,c**) Leaf explant after culture for 3 or 4 weeks, respectively showing primordial somatic embryo cell clumps (arrows) on medium supplemented with 25 μM TDZ. (**d**) Globular stage somatic embryos (arrows) were more visible on the leaf after culture for 5 weeks on medium supplemented with 25 μM TDZ. (**e**) A torpedo stage somatic embryo with a root apical meristem (black arrow) induced at a low concentration (5 μM) of TDZ. (**f**) Torpedo and heart stage somatic embryos (arrows) were visible on the surface of the leaf after culture for 6 weeks. (**g**) Heart stage somatic embryos (arrows) separated from surrounding callus after culture for 7 weeks on medium supplemented with 25 μM TDZ. Bars = 0.2 mm.

The *in vitro* leaves consisted of 6–8 layers of mesophyll cells (Fig. 4a). Exposure to 25 μM TDZ for 4 weeks revealed no obvious changes to the leaf surface. In fact, from the third to fourth week, the meristematic tissue cells swelled and divided into 10–20 layers of cells. Both protuberances without somatic embryos as well as darkly stained embryogenic cell masses developed (Fig. 4b,c). Several globular somatic embryos were observed on the surface of one side of leaf explants, occasionally on both sides, after 5 weeks (Fig. 4d). Globular-shaped somatic embryos developed into heart- or torpedo-shaped somatic embryos after 6–7 weeks (Fig. 4e). Some somatic embryos separated easily from surrounding callus (Fig. 4f). On induction medium supplemented with 25 μM TDZ, no roots were induced due to the high concentration of this cytokinin. Consequently, no root apical meristems were observed. However, somatic embryos were easily separable from leaf sections (Fig. 4f), serving as another distinct difference between somatic embryos and adventitious shoots.

### Root formation and acclimatization of plantlets recovered from shoot and somatic embryo

**Figure 5:**
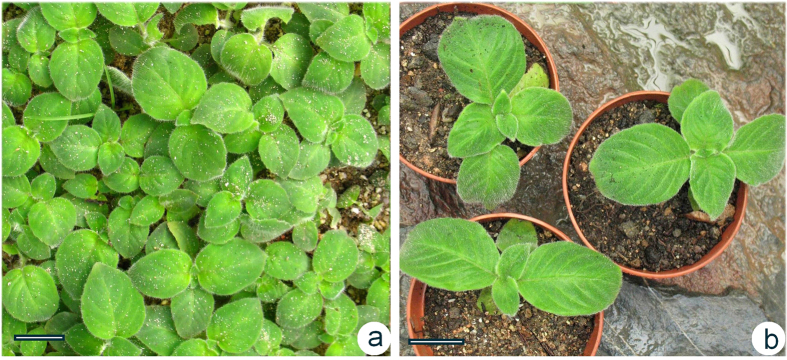
Plant acclimatization, transplantation and the detection of a mutant flowering plant of *Metabriggsia ovalifolia*. (**a**) Two-month-old plants. (**b**) Six-month-old plants. Bars = 2 cm.

Clusters of shoots were divided into individual shoots and transferred to rooting media and rooting was most rapid when exposed to 20 days of continuous light. After 50 days, plantlets with a well-established root system were transferred to a substrate of sand and vermiculite (1:1) leading to more than 95% survival without any obvious morphological variation. Root formation was induced within two weeks as the somatic embryos with an epicotyl were transferred to the rooting medium supplemented with 0.5 μM NAA, 0.5 μM IBA and 0.1% activated charcoal. After plantlets were cultured for another four weeks in rooting medium, they grew to 4–5 cm in height with 4–6 leaves (Fig. 5a). When plantlets were transferred to a sand and vermiculite substrate, more than 93% of the plantlets survived, all but one showing a normal (wild type) phenotypic appearance (Fig. 4a,b).

## Discussion

In a previous report, we established a regeneration system through shoot organogenesis for *M. ovalifolia*^5^. To date, somatic embryogenesis has never been reported for this plant. Here, we used a high concentration of BA and TDZ to successfully induce both shoot organogenesis and somatic embryogenesis. Among the different PGRs whose low concentration (2.5 μM) in induction medium was tested, TDZ and BA induced a high frequency of shoot organogenesis. Auxins, including IAA, IBA and NAA, also induced a low frequency of shoot organogenesis, a response also observed on PGR-free MS medium. However, highly active auxin (2,4-D) and weaker cytokinins (KIN and ZEA) could not induce shoot organogenesis. As BA and TDZ concentration increased, the frequency of somatic embryogenesis increased (Table 2). Moreover, as TDZ concentration reached 25 μM, only somatic embryogenesis was induced (Fig. 2c,d). Similarly, TDZ was the most effective among four cytokinins for shoot regeneration in *Saintpaulia ionantha* (H. Wendl.), but often resulted in poorer shoot quality than BA^9^. As observed for *M. ovalifolia* in this study, *S. ionantha* formed shoots at a low concentration (2.5 μM) of TDZ but somatic embryogenesis occurred at higher concentrations (5–10 μM)^10^.

Most tissue culture studies in the Gesneriaceae family have employed leaf explants, which appear to have the most responsive tissues. Somatic embryogenesis has been successfully induced in some members of the Gesneriaceae: in *Aeschynanthus radicans* Jack ‘Mona Lisa’, globular somatic embryos formed directly from the cut edges of leaf explants as well as from the cut ends on the surface of stem explants 4 weeks after culture on MS medium supplemented with TDZ and NAA, TDZ and 2,4-D, or BA or KN and 2,4-D. Among these, 71% of stem explants produced somatic embryos in response to 9.08 μM TDZ and 2.68 μM 2,4-D. In contrast, 40% of leaf explants induced somatic embryogenesis when MS induction medium was supplemented with 6.81 μM TDZ and 2.68 μM 2,4-D^11^. For *Primulina tabacum* Hance, 5.0 μM of TDZ induced somatic embryos, 5.0 μM of BA induced adventitious shoots while a combination of TDZ and BA in MS medium induced both somatic embryos and adventitious shoots from leaf explants^12^. In that study, adventitious shoots formed when leaf explants were exposed to 5.0 μM TDZ for 30 days then to 5.0 μM BA, but somatic embryos formed when exposed to 5.0 μM BA for 30 days then 5.0 μM TDZ. These two organogenic pathways could be alternated simply by changing the order of the cytokinins (Yang *et al*. 2013). By simply changing the TDZ concentration, Mithila *et al*.^10^ could alter shoot organogenesis to somatic embryogenesis in African violet. In our study, TDZ induced adventitious shoots at a low concentration and somatic embryogenesis at a high concentration. However, BA could only induce both shoot organogenesis and somatic embryogenesis even at higher concentration.

As we used whole leaf explants for culture on induction medium containing 25.0 μM TDZ, we found that the somatic embryos located at the two extremes of leaf explants or in direct contact with medium developed earlier than those somatic embryos developing in central parts of the lamina. This may be related to nutrient absorption and also transfer and metabolism of TDZ. In the former case, the medium could easily transfer and metabolize TDZ while transfer to the central part or metabolism within it could be difficult; consequently, in the central part, somatic embryos remained at an earlier stage, i.e., the globular stage. From another perspective, TDZ induced somatic embryogenesis but also inhibited the regeneration of somatic embryos. This is the first report, to our knowledge, in the Gesneriaceae in which a high concentration of TDZ enhanced somatic embryogenesis while inhibiting the regeneration of somatic embryos. A similar result was achieved with 1–4 mg/l 2,4-D, which induced somatic embryogenesis in cassava, in which 2,4-D enhanced the induction of somatic embryogenesis but also inhibited the regeneration of somatic embryos^13^,^14^. At a high concentration, both 2,4-D and TDZ serve as herbicides^15^,^16^.

Some PGRs are herbicides, including 2,4-D, TDZ, picloram and dicamba, are popular in plant tissue culture studies, and when added to medium at high concentrations, may kill plant tissues and cause developmental aberrations. TDZ is traditionally used as a herbicide in agriculture but became increasingly employed in plant tissue culture^17^. TDZ affects plant physiology, such as cellular, energy, nutrient, transport and metabolic alterations to the membrane, and in flowering *Dendrobium*, alters the endogenous levels of PGRs^18^. The number of biological (physiological and biochemical) events in cells are induced or enhanced by TDZ, but the mode of action of TDZ remains unknown^19^. One somaclonal variant that showed aberrant morphology, and flowering behavior was discovered with the emergence of leaf-like structures from anthers and petals, suggesting that genetic changes may be taking place, which may have formed in response to a high concentration of TDZ or a long subculture period *in vitro*^20^. Another possible reason may be that regeneration from somatic embryos may have the tendency of forming higher levels of somaclonal variation than shoots. This phenomenon of aberrant flowering has not been observed in the previous test with low concentration of TDZ or BA in *M. ovalifolia*^5^, which indicates that a high concentration of TDZ in tissue culture medium may lead to aberrations in *M. ovalifolia*.

## Additional Information

**How to cite this article**: Yao, O. *et al*. Somatic embryogenesis and enhanced shoot organogenesis in *Metabriggsia ovalifolia* W. T. Wang. *Sci. Rep*. **6**, 24662; doi: 10.1038/srep24662 (2016).

## Figures and Tables

**Table 1:** Effect of PGRs on shoot organogenesis from leaf explants of *Metabriggsia ovalifolia*.

PGRs (μM)	Mean number of adventitious shoots induced per explant
No PGRs	1.9 ± 0.9 d
2,4-D 2.5	0 ± 0 e
NAA 2.5	4.6 ± 1.2 c
IBA 2.5	5.7 ± 1.4 c
IAA 2.5	6.4 ± 1.7 c
KIN 2.5	0 ± 0 e
ZEA 2.5	0 ± 0 e
BA 2.5	19.1 ± 2.3 b
TDZ 2.5	31.2 ± 3.1 a

Values within a column followed by different letters indicate significant differences according to the LSD test at *P* < 0.05. All treatments consisted of 30 explants equally divided between five jars and the experiments were repeated three times within a two-week interval.

**Table 2:** Effect of different concentrations of cytokinins (BA and TDZ) on somatic embryogenesis and shoot organogenesis from leaf explants of *Metabriggsia ovalifolia*.

PGRs (μM)	Mean number of induced adventitious shoots per explant	Mean number of induced somatic embryos per explant
BA 1.0	15.4 ± 2.1 b	0 ± 0 e
BA 2.5	19.1 ± 2.4 b	0 ± 0 e
BA 5.0	22.3 ± 2.3 b	3.7 ± 0.6 d
BA 10.0	29.4 ± 3.3 a	4.2 ± 1.2 d
BA 25.0	23.6 ± 2.5 b	9.7 ± 1.6 d
TDZ 1.0	18.9 ± 2.7 b	0 ± 0 e
TDZ 2.5	31.2 ± 3.5 a	0 ± 0 e
TDZ 5.0	21.4 ± 2.3 b	26.5 ± 2.4 c
TDZ 10.0	9.7 ± 1.5 c	50.3 ± 3.5 a
TDZ 25.0	0 ± 0 e	39.8 ± 3.1 b

Data within a column followed by different letters indicate significantly differences according to the LSD test at *P* < 0.05. All treatments consisted of 30 explants equally divided between five jars and the experiments were repeated three times within a two-week interval.

## References

[b1] Tan Y. H. *Metabriggsia* WT Wang, a newly recorded genus of Gesneriaceae from Yunnan, China. Acta Bot. Boreal. – Occident. Sin. 32, 2122–2123 (2012) (in Chinese with English abstract).

[b2] Wang W. T. Genus novum Gesneriacearume Guangxi. Guihaia 1, 1–6 (1983) (in Chinese).

[b3] Lu Y. X., Huang G. B. & Liang C. F. Study on the endemic plants from Guangxi. Guihaia 9, 37–58 (1989) (Chinese).

[b4] Wang S. & Xie Y. China Species Red List (Vol. 1): Red List. Beijing, Higher Education Press, pp. 240–241 (2004) (in Chinese).

[b5] Ma G. H., Teixeira da Silva J. A., Lü J. F., Zhang X. H. & Zhao J. T. Shoot organogenesis and plant regeneration in *Metabriggsia ovalifolia*. Plant Cell Tiss. Org. Cult. 105, 355–361 (2011).

[b6] Yang X. Y., Lü J. F., Teixeira da Silva J. A. & Ma G. H. Somatic embryogenesis and shoot organogenesis from leaf explants of *Primulina tabacum*. Plant Cell Tiss. Org. Cult. 109, 213–221 (2012).

[b7] Murashige T. & Skoog F. A revised medium for rapid growth and bioassays with tobacco tissue cultures. Physiol. Plant. 15, 473–497 (1962).

[b8] Lü J.-F., Teixeira da Silva J. A. & Ma G. H. Plant regeneration via somatic embryogenesis and shoot organogenesis from immature cotyledons of *Camellia nitidissima*. J. Plant Physiol. 170, 1202–1211 (2013). 23790533 10.1016/j.jplph.2013.03.019

[b9] Winkelmann T. & Grunewaldt J. Genotypic variability for protoplast regeneration in *Saintpaulia ionantha* (H. Wendl.). Plant Cell Rep. 14, 704–707 (1995). 24186626 10.1007/BF00232651

[b10] Mithila J., Hall J. C., Victor J. M. R. & Saxena P. K. Thidiazuron induces shoot organogenesis at low concentrations and somatic embryogenesis at high concentrations on leaf and petiole explants of African violet (*Saintpaulia ionantha* Wendl.). Plant Cell Rep. 21, 408–414 (2003). 12789442 10.1007/s00299-002-0544-y

[b11] Cui J., Chen J. J. & Henny R. Regeneration of *Aeschynanthus radicans* via direct somatic embryogenesis and analysis of regenerants with flow cytometry. In Vitro Cell. Dev. Biol. - Plant 45, 34–43 (2009).

[b12] Ma G. H., Teixeira da Silva J. A. Lü J. F. Zhang X. H. & Zhao J. T. Direct somatic embryogenesis and shoot organogenesis from leaf explants of *Primulina tabacum* Hance. Biol. Plant. 54, 361–365 (2010).

[b13] Ma G. H. & Xu Q. S. Induction of somatic embryogenesis and adventitious shoot formation from immature leaves of cassava. Plant Cell Tiss. Org. Cult. 70, 281–288 (2002).

[b14] Ma G. H. Effect of cytokinins and auxins on cassava somatic embryogenesis and shoot organogenesis from somatic embryos. Plant Cell Tiss. Org. Cult. 54, 1–7 (1998).

[b15] Peterson M. E. & Talcott P. A. Small Animal Toxicology. 2nd ed; Saunders Elsevier: St. Louis, pp 734–735 (2006).

[b16] Potter T. L., Marti L., Belflower S. & Truman C. C. Multiresidue analysis of cotton defoliant, herbicide, and insecticide residues in water by Solid-Phase Extraction and GC−NPD, GC−MS, and HPLC−Diode Array Detection. J. Agric. Food Chem. 48, 4103–4108 (2000). 10995322 10.1021/jf9909104

[b17] Srinivasan M., Nachiappan V. & Rajasekharan R. Potential application of urea-derived herbicides as cytokinins in plant tissue culture. J. Biosci. 31, 599–605 (2006) 17301498 10.1007/BF02708412

[b18] de Melo Ferreira W., Kerbauy G. B., Kraus J. E., Pescador R. & Mamoru Suzuki R. Thidiazuron influences the endogenous levels of cytokinins and IAA during the flowering of isolated shoots of *Dendrobium*. J. Plant Physiol. 163, 1126–1134 (2006). 17032618 10.1016/j.jplph.2005.07.012

[b19] Guo B., Abbasi B. H., Zeb A., Xu L. L. & Wei Y. H. Thidiazuron: A multi-dimensional plant growth regulator. African J. Biotech. 10, 8984–9000 (2011).

[b20] Zhang C. G. Li W., Mao Y. F., Zhao D. L. & Dong W. Endogenous hormonal levels in *Scutellaria baicalensis* calli induced by thidiazuron. *Russ*. J. Plant Physiol. 52, 345–351 (2005).

